# Road Traffic Noise, Air Pollutants, and the Prevalence of Cardiovascular Disease in Taichung, Taiwan

**DOI:** 10.3390/ijerph15081707

**Published:** 2018-08-09

**Authors:** Wei-Ting Yang, Ven-Shing Wang, Li-Te Chang, Kai-Jen Chuang, Hsiao-Chi Chuang, Chiu-Shong Liu, Bo-Ying Bao, Ta-Yuan Chang

**Affiliations:** 1 Department of Occupational Safety and Health, College of Public Health, China Medical University, No. 91, Hsueh-Shih Road, Taichung 40402, Taiwan; angela24682468@hotmail.com (W.-T.Y.); vswang@mail.cmu.edu.tw (V.-S.W.); 2 Department of Environmental Engineering and Science, Feng Chia University, No. 100, Wen-Hwa Road, Taichung 40724, Taiwan; ltechang@fcu.edu.tw; 3 Department of Public Health, School of Medicine, College of Medicine, Taipei Medical University, No. 250, Wu-Hsing Street, Taipei 11031, Taiwan; kjc@tmu.edu.tw; 4 School of Public Health, College of Public Health and Nutrition, Taipei Medical University, No. 250, Wu-Hsing Street, Taipei 11031, Taiwan; 5 School of Respiratory Therapy, College of Medicine, Taipei Medical University, No. 250, Wu-Hsing Street, Taipei 11031, Taiwan; chuanghc@tmu.edu.tw; 6 Department of Internal Medicine, School of Medicine, College of Medicine, Taipei Medical University, No. 250, Wu-Hsing Street, Taipei 11031, Taiwan; 7 Department of Family Medicine, China Medical University Hospital, Taichung 40447, Taiwan; liucs@ms14.hinet.net; 8 Department of Pharmacy, College of Pharmacy, China Medical University, No. 91, Hsueh-Shih Road, Taichung 40402, Taiwan; bao@mail.cmu.edu.tw; 9 Department of Nursing, Asia University, No. 500, Lioufeng Road, Wufeng, Taichung 41354, Taiwan

**Keywords:** air pollutants, cardiovascular disease, prevalence, road traffic noise

## Abstract

*Background:* A few studies have investigated the interaction between exposure to road traffic noise, air pollutants, and cardiovascular disease (CVD), but their results were inconsistent. This cross-sectional study investigated whether road traffic noise, particulate matter with dynamic diameter less than 10 μm (PM_10_) and nitrogen dioxides (NO_2_) exposure were independently associated with the risk of CVD. *Methods*: We recruited 663 volunteers who had been living near main roads for more than three years in 2008. Information concerning the subjects’ home addresses was combined with noise measurements at 42 locations and annual average of air pollutants from 2 monitoring stations to estimate individual exposure. Multivariate logistic regression was used to calculate the odds ratio (OR) for diagnosed CVD, adjusting for potential confounders and co-exposure. *Results*: Only per 5-dBA increase in road traffic noise was significantly associated with elevated risk of CVD (adjusted OR = 2.23, 95% confidence interval (CI) = 1.26–3.93) in the single-exposure models. Such association was aggravated (adjusted OR = 2.96, 95% CI = 1.41–6.23) after adjustment for total traffic and PM_10_ or NO_2_ in the two-exposure models. *Conclusions*: Road traffic noise exposure may be associated with the increasing prevalence of CVD. No synergistic association was observed between co-exposure to noise and air pollutants and the risk of CVD.

## 1. Introduction

Road traffic is the main source for noise and ambient air pollutant exposure in metropolitan areas. Beside the auditory effects of noise exposure on health (i.e., annoyance, disturbing sleep and noise-induced hearing loss), the non-auditory effects (such as cardiovascular disease (CVD) and impaired cognition in school children) are concerns for the public health [1,2]. Many environmental epidemiological studies have reported the association between exposure to noise or air pollutants and morbidity and mortality of CVD. van Kempen and Babisch have estimated the relationship between road traffic noise and hypertension in a meta-analysis study [3]. Vienneau and his colleagues further presented an exposure-response relationship between transportation noise exposure and ischemic heart disease [4]. In addition, Brook et al. updated a scientific statement to conclude a causal relationship between exposure to particulate matter with dynamic diameter less than 2.5 μm (PM_2.5_) and cardiovascular morbidity and mortality from the American Heart Association [5]. Liang and his colleagues conducted a systematic review to report the significant impact of PM_2.5_ exposure on blood pressure, with the strongest association for the long-term exposure [6]. A meta-analysis study further showed the scientific evidence that long-term exposure to particulate matter with dynamic diameter less than 10 μm (PM_10_) is regarded as a risk factor of stroke [7]. Mannsaker et al. reported that exposure to nitric oxide (NO) and nitrogen dioxides (NO_2_) may influence the hospitalizations for cardiovascular disease [8]. Liu and his colleagues also found that exposure to NO_2_ was significantly associated with CVD mortality [9].

A few studies have investigated the interaction between exposure to road traffic noise, air pollutants, and CVD, but their results were inconsistent. A cohort study observed the increased risks of cerebrovascular and heart failure mortality for elevated levels of black smoke after adjustment for traffic noise, but the excess of ischemic heart disease (IHD) and heart failure mortality in the highest noise category (>65 A-weighted decibel (dBA)) reduced to unity after controlling for black smoke and traffic intensity [10]. In a multi-national study, 24-h average road traffic noise exposure was associated with heart disease and stroke, but adjustment for NO_2_ in the subsample suggested this may have been due to confounding by air pollution [11]. In a time-stratified case-crossover design, per 1-dBA increase in maximum nocturnal noise levels was significantly associated with the increased mortality for IHD and myocardial infarction after adjustment for PM_2.5_, NO_2_, mean temperature, and relative humidity [12]. The reasons for these inconsistencies may be differences in the study design, exposure assessment, and adjustments for potential confounders. Because exposure to noise or air pollutants was reported to be associated with elevated risk of CVD, we hypothesized a synergistic effect of co-exposure to noise and air pollution. This cross-sectional study aimed to investigate whether road traffic noise, PM_10_, and NO_2_ exposure were independently associated with the prevalence of CVD among residents in Taichung, Taiwan.

## 2. Materials and Methods

### 2.1. Study Areas and Population

This study was performed in Taichung city with a population of 1.07 million people located in central Taiwan. The detailed design was reported in previous studies [13,14]. Briefly, four main roads that cover 77.3% of inhabitants were chosen as study areas, including three roads radiating from the Taichung Station across the city and one road linked to the other roads near this station. A total of 42 sampling sites were established at 1-km intervals radiating from the station to measure road traffic noise levels and traffic flow rates simultaneously. We recruited 20 households for each sampling site along the four main roads during June to September in 2008. Only one person per household was invited to join in this study. Each subject has to live within a 100-m radius of these sampling sites over three years at the current address. The face-to-face interview was conducted by four well-trained investigators at subjects’ homes during the periods of road traffic noise measurements. Because this study was carried out from Tuesday to Thursday, most of the participants are retailers, owners of grocery stores, housewives, and retired inhabitants living in the shopping district and residential district of Taichung City. We excluded 20 subjects who had lived in their residences for less than 3 years and 120 subjects who did not live at the current address. Ultimately, 663 residents were included as study subjects. No significant differences in mean age; body mass index (BMI); cigarette smoking; alcohol, tea, and coffee consumption; salt intake; and family history of hypertension were identified between 663 participants and 140 non-participants (all *p* values > 0.05). The Institutional Review Board of the School of Public Health of China Medical University has reviewed and approved this study design before its start, and each subject has provided written informed consent to participate in this study.

### 2.2. Questionnaire and Definition of Cardiovascular Disease

A standardized questionnaire was applied to obtain personal data related to potential risk factors of CVD. These factors included age, gender, height, weight, cigarette smoking, alcohol drinking, tea and coffee consumption, daily salt intake, exercise habits, and family history of hypertension. All subjects’ lifestyle habits were defined in detail to avoid information bias. Current cigarette smokers were defined as subjects who admitted to smoking at least three times per week for more than six months. Similar definitions were used to classify current alcohol drinkers, and tea and coffee consumers. Participants were considered to have high salt intake if they reported that they consumed more dietary salt than other study subjects based on the median consumption. Regular exercisers were defined as those who participated in exercise at least three times per week for six months or more. A family history of hypertension was positive if the subject reported to have parents or grandparents with doctor-diagnosed hypertension. Additionally, body weight (kg) was divided with the square of the height (m^2^) to calculate body mass index (BMI) for each subject.

Participants were regarded as a case of CVD if he/she answered affirmatively to the question: “Have you been diagnosed with CVD by a physician in the past while living at the current address?” Among the 663 subjects in this study, 46 cases were identified using this criterion. Accordingly, the participants were classified into a case group of 46 subjects and a control group of the remaining 617 subjects.

### 2.3. Road Traffic Noise Measurements and Traffic Calculations

Road traffic noise levels were determined using an octave-band analyzer (TES-1358, TES Electronic Corp., Taipei, Taiwan), which can report 1-s to 24-h continuous equivalent sound levels (Leq) in the range of 30–130 A-weighted decibels (dBA) and time-weighted average (TWA) noise levels. Before conducting the noise measurements, a sound-level calibrator (TES-1356, TES Electronic Corp., Taipei, Taiwan) was used to calibrate this equipment. We set up 42 sampling sites at 1-km intervals along each of four main roads, and located these sampling sites 1 m away from buildings with a height of 1.5 m from the ground. Two industrial hygienists measured 15-min TWA Leq at each sampling site on weekdays from 9:00–17:00. All subjects were divided into 42 groups based on the closest sampling site to assign their daily exposure to road traffic noise levels. The distance between the sampling site and a subject’s address ranged from 5.2 m to 67.7 m within each group. Each participant was assigned to one value of road traffic noise exposure that corresponded with the 8-h TWA Leq (L_Aeq,8h_) measured at the closest site.

During the monitoring period, two research assistants evaluated traffic flow rates of heavy-duty diesel trucks (≥3.5 ton), light-duty diesel trucks (<3.5 ton), light-duty gasoline vehicles (<3.5 ton), and motorcycles at each of 42 sampling sites. Each assistant was responsible for calculating two types of traffic vehicles passing in front of the sampling sites. The sum of motorcycles, light-duty gasoline vehicles, light-duty diesel trucks, and heavy-duty diesel trucks was used for total traffic flow rate in this study.

### 2.4. Air Pollutants Monitoring Levels

**Figure 1:**
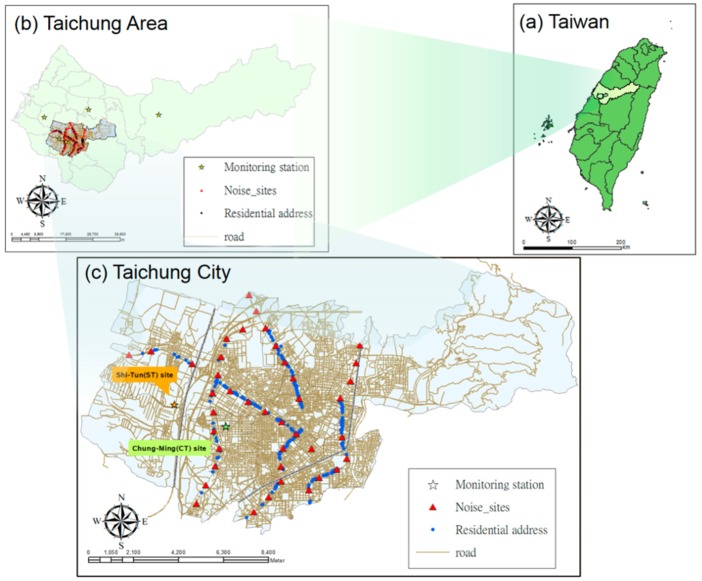
Locations of noise, air quality monitoring stations, and subjects’ house addresses. (**a**) Taiwan; (**b**) Taichung area; (**c**) Taichung city.

We used the geographic information system (GIS) software in ArcGIS 9.3.1 (Environmental Systems Research Institute Incorporated, Redlands, CA, USA) to identify all participants’ home addresses and the locations of five air quality monitoring stations in Taichung. These monitoring stations were established around the study area by the Taiwan Environmental Protection Administration (Taiwan EPA). Because all subjects lived in Taichung City, only air quality data at two monitoring stations (i.e., Chung-Ming [CM] site and Shi-Tun [ST] site) were used in this study. The distances between individual home addresses and these two monitoring stations ranged between 0.5–5.7 km from the CM site and 1.6–8.5 km from the ST site, as shown in Figure 1.

We adopted the following equation to estimate annual average levels of specific air pollutants for individuals based on residing distance to both monitoring stations [15]:

(1) $$\frac{mean\;in\;CM\;site\times distance\;to\;ST\;site+mean\;in\;ST\;site\times distance\;to\;CM\;site}{distance\;to\;CM\;site+distance\;to\;ST\;site}$$

Hourly monitoring data on PM_10_ and NO_2_ levels over 24 h in 2008 were collected and calculated to have an annual average for each site. All participants were assigned to exposure levels of air pollutants based on the above equation.

### 2.5. Statistical Analysis

We first used the Shapiro–Wilk test to determine the normality of the continuous variables, including age, BMI, L_Aeq,8h_, PM_10_, and NO_2_ levels. Because statistical *p* values for these variables were less than 0.001 among all participants that showed non-normal distribution, the Wilcoxon signed rank sum test was performed to examine univariate comparisons between different groups for continuous variables. In addition, the Chi-square test was applied to compare the differences between groups for dichotomous variables. The Spearman rank correlation was used to investigate the correlations between road traffic noise, air pollutants, and total traffic among subjects.

Continuous (i.e., 5-dBA increase in road traffic noise, 1 µg/m^3^ increase in PM_10_, and 1 ppb increase in NO_2_) and categorical variables (i.e., high-exposure group versus low-exposure group) of road traffic noise and air pollutants among participants were used to examine the association with the prevalence of CVD. We used 80 dBA as the cut-off value for noise exposure categories because exposure to L_Aeq,8h_ ≥ 80 dBA has been reported to be associated with an increased prevalence of hypertension among residents [13]. The median value for PM_10_ (58 µg/m^3^) was used to divide subjects into high- and low-exposure groups with a similar number in each subgroup, because participants being exposed to PM_10_ (ranged 57–59 μg/m^3^) levels was higher than air quality annual guidelines of the World Health Organization (WHO) (PM_10_: 20 μg/m^3^) [16], while still lower than the Taiwan annual standard of air quality (65 μg/m^3^) [17]. We selected 20 ppb as the cut-off value for NO_2_ exposure categories based on the WHO guideline (NO_2_: 40 μg/m^3^, equal to 19.5 ppb at 1 atmosphere, 0 °C) [16]. Accordingly, 663 participants were further separated into co-exposure to high-noise and high-air pollutants group, high-noise and low-air pollutants group, low-noise and high-air pollutants group, and low-noise and low-air pollutants group to investigate the interaction.

We used logistic regression models to calculate odds ratios (ORs) and 95% confidence intervals (CIs) in this study. For each combination of high and low exposure groups for road traffic noise, PM_10_ and NO_2_, the crude OR of self-reported CVD in participants above versus below the median exposure were calculated by simple logistic regression models. We used the following selection process to identify covariates in the best fit model. First, a full model was constructed with the outcome variable of CVD prevalence and all covariates. We applied the forward method with an entry level of 0.10 and identified three variables (i.e., age, BMI, and family history of hypertension) significantly associated with the outcome (all *p* values < 0.05). These covariates were then included in a reduced model. Finally, every possible combination of remaining variables (i.e., gender, cigarette smoking, and alcohol, tea and coffee consumption, regular exercise, salt intake) was added to the reduced model, and the Akaike information criterion (AIC) of these models was compared. Based on this criterion, three variables of age, BMI, and family history of hypertension were kept with the lowest AIC to create the final adjusted model. In addition to these three variables, five important risk factors of CVD reported in the literature [18,19,20], including gender, cigarette smoking, alcohol consumption, salt intake, and physical inactivity, were included in the multivariable logistic regression as the final model. Model selection was repeated for every combination of exposure variables (i.e., dichotomous high- vs. low-exposure groups or four different co-exposure groups) and the outcome to ensure similar selection of covariates.

We also present the ORs and 95% CI to show the risk of CVD prevalence per 5-dBA increase in noise, 1 μg/m^3^ increase in PM_10_, and 1 ppb increase in NO_2_ exposure in the single-exposure models. In order to investigate the interaction between road traffic noise and air pollutants exposure, both noise and PM_10_ (or NO_2_) were included simultaneously in a multivariable logistic regression (two-exposure model) to compare the differences in exposure-effect estimates before and after mutual adjustment. The C statistic value was used to show the concordance between the model estimate and the observed case of CVD [21] and the Hosmer and Lemeshow method was applied for the goodness-of-fit test. C statistic values higher than 0.7 were generally considered fair and clinically useful [21] and the probability of the Hosmer and Lemeshow test greater than 0.05 indicated the goodness of fit for the model. The variance inflation factor (VIF) was used to test the multi-collinearity in the regression models and a VIF value of 3 was selected as the cut-off point to indicate multi-collinearity [22]. Neither of the interaction term of noise and PM_10_ nor of noise and NO_2_ were not included in the two-exposure models because of the severe multi-collinearity (both VIF values > 10). Instead, the total traffic was put in the single-exposure and two-exposure models to control for other air pollutants. We used the SAS standard package for Windows version 9.2 (SAS Institute Incorporation, Cary, NC, USA) to perform statistical analyses and set the significance level at 0.05 for all statistical tests in the present study.

## 3. Results

Table 1 presents the demographic characteristics among all participants. Male subjects had a significantly higher mean of BMI as well as higher proportions of cigarette smoking, alcohol consumption, high salt intake, and regular exercise than female subjects.

Table 2 shows the exposure levels of road traffic noise, PM_10_, NO_2_, and total traffic among study subjects. No significant differences in these exposure variables were identified by gender.

The spearman’s correlations between road traffic noise, air pollutants, and total traffic among subjects are shown in Table 3. Only noise levels were significantly correlated to the total traffic (correlation coefficient = 0.593, *p* < 0.001). No significant correlations between noise and air pollutants indicated that all exposure levels were independent and appropriate for investigating single and multiple main effects.

Table 4 reveals the associations between different exposure groups and the prevalence of cardiovascular disease. The high-noise-exposure group (≥80 dBA) had a marginally higher OR of CVD prevalence (1.95, 95% CI = 0.99–3.85, *p* = 0.053) compared with the low-noise-exposure group (<80 dBA) after adjustment for potential confounders. No significant differences in the prevalence of CVD were observed between the high-exposure and low-exposure subjects exposed to either PM_10_ or NO_2_ levels (both *p* values > 0.05).

The association between co-exposure groups and the prevalence of cardiovascular disease is shown in Table 5. Co-exposure with high noise (≥80 dBA) and high PM_10_ (≥58 µg/m^3^), as well as co-exposure to high noise (≥80 dBA) and high NO_2_ (≥20 ppb), had the higher adjusted OR (2.20, 95% CI = 0.73–6.62; 1.84, 95% CI = 0.79–4.31) compared with the reference groups, but no categorized co-exposure groups were found to be significant (all *p* values > 0.05).

Table 6 presents the associations between continuous exposure of noise and air pollutants and the prevalence of cardiovascular disease. In the single-exposure models, only per 5-dBA increase in road traffic noise was significantly associated with the increased risk of prevalent CVD (adjusted OR = 2.23, 95% CI = 1.26–3.93). In the two-exposure models, per 5-dBA increase in road traffic noise was significantly associated with the 2.22-fold (95% CI = 1.26–3.93) and 2.96-fold (95% CI = 1.41–6.23) ORs of prevalent CVD before and after adjustment for the total traffic flow rate and other confounders. No significant associations were found between the 1-μg/m^3^ increase in PM_10_ or 1-ppb increase in NO_2_ and the prevalence of CVD in both single-exposure and two-exposure models (all *p* values > 0.05). All models with C statistic values higher than 0.8 and *p* values greater than 0.10 in the Hosmer and Lemeshow test indicated the good concordance and well fit of covariates. The VIF values were all less than 2 to show no multi-collinearity between road traffic noise, PM_10_, NO_2_, traffic flow, and other covariates in the multivariable logistic regression models.

## 4. Discussion

This study observed a significant association between road traffic noise exposure and the prevalence of CVD after adjustment for total traffic and PM_10_ or NO_2_. The outcome was consistent with the finding in a previous study that reported the increased prevalence of self-reported hypertension associated with the exposure to road traffic noise levels controlling for traffic flow rate [13]. In contrast, Floud and her colleagues found that the association between 24-h average road traffic noise exposure and “heart disease and stroke” became non-significant after adjustment for NO_2_ in the subsample [11]. Such inconsistency may be due to the difference in precision and accuracy of the noise exposure assessment that was conducted by noise measurements in the current and previous study [13], but by the noise predictive model for the later [11].

In addition, the association between road traffic noise exposure and the prevalence of CVD was easily underestimated because of the interaction of air pollutants. The effect estimate of per 5-dBA increase in road traffic noise exposure on the prevalence of CVD was elevated from the OR of 2.23 in the single-exposure model to the value of 2.96 after the consideration of total traffic and PM_10_ (or NO_2_) in the two-exposure models. The phenomenon indicated the potential interaction between road traffic noise and other air pollutants (such as black smoke, PM_2.5_, NO, SO_2_, etc.) in the metropolitan areas. Future studies should take total traffic into account, while investigating the independent associations between the prevalence of CVD and exposure to road traffic noise, PM_10_, NO_2_, or other air pollutants.

In the current study, we did not observe significant associations between the prevalence of CVD and exposure to PM_10_ or NO_2_ levels before and after adjustment for road traffic noise exposure. In contrast, Beelen et al. reported increased risks of cerebrovascular and heart failure mortality for elevated levels of black smoke after controlling for traffic noise [10]. Liu and his colleagues also found an increased CVD mortality for exposure to NO_2_, but not for PM_10_ [9]. Because of the differences in study design (two cohort studies vs. this cross-sectional study), exposure assessment (e.g., predictive models vs. air monitoring stations, representativeness of monitoring stations) and adjustments for other exposure variables (i.e., road traffic noise) between these studies, more research is needed to elucidate the association between CVD and exposure to air pollutants.

In addition, we did not find significantly synergistic effects of co-exposure to noise and air pollution in the present study. We classified subjects into high- and low-exposure groups based on noise, PM_10_, and NO_2_ levels and compared the ORs in one-high (i.e., noise, PM_10_, or NO_2_) or two-high exposure items (i.e., noise and PM_10_ or noise and NO_2_) with the reference group. Most high-exposure groups (either one or two exposure items) had an increased but non-significant risk of CVD prevalence compared with the low-exposure groups, except for the high-NO_2_ and low-noise exposure group (OR < 1). One possible reason is that subjects were exposed to too high levels of noise (range: 73.9–85.4 dBA), PM_10_, (range: 57.6–59.3 μg/m^3^) and NO_2_ (range: 19.1–21.1 ppb) compared with the WHO guidelines (e.g., 65 dBA, 15 μg/m^3^, and 19.5 ppb, respectively) to observe the significant difference [16,23]. The other reason is a too low number of cases in each group to reach the statistical differences. Future studies are encouraged to overcome these limitations.

The WHO guidelines for community noise is set up at 65 dBA over 24 h to prevent CVD among general population [23]. The 8-h average A-weighted equivalent noise level (L_Aeq,8h_) in this study was 79.4 ± 3.2 dBA, which was higher than the WHO criterion to observe the elevated risk among residents. Compared with the findings of previous studies, the daytime noise levels in Taichung was higher than those measured for 10 min during 3 months in Atlanta (65.1 ± 7.4 dBA), Los Angeles (66.4 ± 4.64 dBA), and New York (69.2 ± 4.1 dBA), in the United States [24], as well as those monitored for 20 min in non-rush hours during weekdays in three European Cities of Basel, Switzerland (61.1 ± 6.4 dBA); Girona, Spain (63.9 ± 6.2 dBA); and Grenobel, France (64.5 ± 6.7 dBA) [22]. Because the road traffic noise levels are mainly generated from traffic vehicles, we recommend that the relevant governmental agencies have to take actions immediately for reducing the noise levels to prevent the CVD risk.

The strength of this study is the precise and accurate exposure assessment of road traffic noise exposure and the objective estimation of air-pollution exposure through the use of air monitoring stations. These advantages give the opportunity to investigate the association between co-exposure to road traffic noise and air pollutants and the risk of CVD. However, some limitations have to be mentioned. The major restriction is the cross-sectional design that cannot explain the causal relationship between exposure and disease. The second is the use of self-reported CVD instead of diagnosed outcome, which may produce an underestimation of CVD cases in the present study. The third is not considering noise perception and noise sensitivity for susceptible subjects. The fourth is that measurements of air pollutants in two monitoring stations are not co-located with the noise measurements to completely represent roadside exposures. The fifth is the use of one annual average to represent subjects’ exposure to air pollutants. The sixth is that only 46 CVD cases identified in this study may over-stratify the data to produce the non-significant results as very few cases for some categories. Finally, some confounders related to CVD are not collected here, including total cholesterol, triglyceride, and glucose levels [18,19,20].

## 5. Conclusions

This study observed the significant association between road traffic noise exposure and the prevalence of CVD with regards to these limitations. Exposure to PM_10_ and NO_2_ was not found to increase the prevalence of CVD, before or after controlling for road traffic noise levels. Because exposure levels of noise and air pollutants were higher than WHO guidelines, and a very low number of CVD cases were included, no significantly synergistic effects of co-exposure were reported in this study. Future studies have to overcome the differences in study design, exposure assessment, and adjustments for potential confounders to prove the findings in the current study. In addition, the road traffic noise exposure in Taichung was higher than the WHO criterion and other cities in the Europe and America. The relevant governmental agencies in Taiwan are obligated to generate strategies for mitigating noise levels to prevent the CVD risk in public health.

## Figures and Tables

**Table 1:** Demographic characteristics of participants.

Characteristics	Male	Female	Total	*p*-Value
No. of subjects	256	407	663	
Age [(years (mean ± SD)] ^a^	42.4 (23.0)	45.9 (26.7)	44.5 (25.4)	0.3710
BMI [(kg/m^2^(mean ± SD)] ^a^	23.7 (3.7)	21.7 (3.6)	22.5 (3.8)	<0.0001
Smoking [no. (%)] ^b^				<0.0001
Yes (%)	102 (39.8)	41 (10.1)	143 (21.6)	
No (%)	154 (60.2)	366 (89.9)	520 (78.4)	
Alcohol drinking [no. (%)] ^b^				<0.0001
Yes (%)	51 (19.9)	18 (4.4)	69 (10.4)	
No (%)	205 (80.1)	389 (95.6)	594 (89.6)	
Tea consumption [no. (%)] ^b^				0.4774
Yes (%)	161 (62.9)	267 (65.6)	428 (64.6)	
No (%)	95 (37.1)	140 (34.4)	235 (35.4)	
Coffee consumption [no. (%)] ^b^				0.0697
Yes (%)	84 (32.8)	162 (39.8)	246 (37.1)	
No (%)	172 (67.2)	245 (60.2)	417 (62.9)	
Salt intake [no. (%)] ^b^				0.0309
High (%)	75 (29.3)	89 (21.9)	164 (24.7)	
Low (%)	181 (70.7)	318 (78.1)	499 (75.3)	
Regular exercise [no. (%)] ^b^				<0.0001
Yes (%)	147 (57.4)	166 (40.8)	313 (47.2)	
No (%)	109 (42.6)	241 (59.2)	350 (52.8)	
Family history of hypertension [no. (%)] ^b^				0.2861
Yes (%)	76 (29.7)	137 (33.7)	213 (32.1)	
No (%)	180 (70.3)	270 (66.3)	450 (67.9)	

SD, standard deviation; ^a^ Wilcoxon rank sum test of difference between male and female; ^b^ Chi-square test of difference between male and female. BMI—body mass index.

**Table 2:** Road traffic noise and air pollutants among study subjects.

	Male	Female	Total	*p*-Value
No. of subjects	256	407	663	
L_Aeq,8h_ (dBA) ^a^				0.7422
Mean (SD)	79.5 (3.3)	79.4 (3.1)	79.4 (3.2)	
Range	73.9–85.4	73.9–85.4	73.9–85.4	
Total traffic (vehicle/day) ^a^				0.7842
Mean (SD)	434 (221)	442 (200)	438 (208)	
Range	82–794	82–794	82–794	
PM_10_ (µg/m^3^) ^a^				0.0898
Mean (SD)	58.4 (0.3)	58.3 (0.3)	58.3 (0.3)	
Range	57.6–59.3	57.6–59.3	57.6–59.3	
NO_2_ (ppb) ^a^				0.0898
Mean (SD)	20.2 (0.4)	20.3 (0.3)	20.2 (0.3)	
Range	19.2–21.1	19.1–21.1	19.1–21.1	

dBA, A-weighted decibel; L_Aeq,8h_, A-weighted equivalent sound level at 9:00–17:00; PM_10_, particulate matter less than 10 μm with aerodynamic diameter; NO_2_, nitrogen dioxides; SD, standard deviation; ^a^ Wilcoxon rank sum test of difference between male and female.

**Table 3:** Spearman’s correlations between noise, air pollutants, and traffic in subjects.

	PM_10_ (μg/m^3^)	NO_2_ (ppb)	Total Traffic (vehicle/day)
L_Aeq,8h_ (dBA)	0.068	−0.068	0.593
*p*-value	0.0789	0.0789	<0.0001

dBA, A-weighted decibel; L_Aeq,8h_, A-weighted equivalent sound level at 9:00–17:00; PM_10_, particulate matter less than 10 μm with aerodynamic diameter; NO_2_, nitrogen dioxides.

**Table 4:** Associations between single-exposure groups and the prevalence of cardiovascular disease.

	CVD Case [no. (%)]	Crude OR (95% CI) ^a^	Adjusted OR (95% CI) ^b^
Model 1			
High-exposure (noise level ≥ 80 dBA, n = 313)	27 (8.63)	1.65 (0.90–3.02)	1.95 (0.99–3.85)
Low-exposure (noise level < 80 dBA, n = 350)	19 (5.43)	1.00	1.00
Model 2			
High-exposure (PM_10_ level ≥ 58 μg/m^3^, n = 331)	22 (6.65)	0.91 (0.50–1.66)	0.94 (0.49–1.81)
Low-exposure (PM_10_ level < 58 μg/m^3^, n = 332)	24 (7.23)	1.00	1.00
Model 3			
High-exposure (NO_2_ level ≥ 20 ppb, n = 334)	24 (7.29)	1.12 (0.61–2.03)	1.08 (0.56–2.08)
Low-exposure (NO_2_ level < 20 ppb, n = 329)	22 (6.59)	1.00	1.00

CVD, cardiovascular disease; dBA, A-weighted decibel; PM_10_, particulate matter less than 10 μm with aerodynamic diameter; NO_2_, nitrogen dioxides; OR, odds ratio; 95% CI, 95% confidence interval; ^a^ simple logistic regression models; ^b^ the multivariable logistic regression was adjusted for age, gender, body mass index, smoking, alcohol consumption, salt intake, regular exercise, and family history of hypertension.

**Table 5:** Associations between co-exposure groups and the prevalence of cardiovascular disease.

	Number	CVD Case [no. (%)]	Crude OR (95% CI) ^a^	Adjusted OR (95% CI) ^b^
Model 1:				
Noise ≥ 80 dBA and PM_10_ ≥ 58 μg/m^3^	140	10 (7.14)	1.67 (0.62–4.51)	2.20 (0.73–6.62)
Noise ≥ 80 dBA and PM_10_ < 58 μg/m^3^	173	17 (9.83)	2.37 (0.95–5.87)	2.45 (0.88–6.82)
Noise < 80 dBA and PM_10_ ≥ 58 μg/m^3^	191	12 (6.28)	1.46 (0.56–3.79)	1.35 (0.47–3.87)
Noise < 80 dBA and PM_10_ < 58 μg/m^3^	159	7 (4.40)	1.00	1.00
Model 2:				
Noise ≥ 80 dBA and NO_2_ ≥ 20 ppb	171	17 (9.94)	1.66 (0.77–3.58)	1.84 (0.79–4.31)
Noise ≥ 80 dBA and NO_2_ < 20 ppb	142	10 (7.04)	1.14 (0.48–2.71)	1.60 (0.61–4.18)
Noise < 80 dBA and NO_2_ ≥ 20 ppb	158	7 (4.43)	0.70 (0.27–1.81)	0.74 (0.26–2.12)
Noise < 80 dBA and NO_2_ < 20 ppb	192	12 (6.25)	1.00	1.00

CVD, cardiovascular disease; dBA, A-weighted decibel; PM_10_, particulate matter less than 10 μm with aerodynamic diameter; NO_2_, nitrogen dioxides; OR, odds ratio; 95% CI, 95% confidence interval; ^a^ simple logistic regression models; ^b^ the multivariable logistic regression was adjusted for age, gender, body mass index, smoking, alcohol consumption, salt intake, regular exercise, and family history of hypertension.

**Table 6:** Associations between continuous exposure of noise and air pollutants and the prevalence of cardiovascular disease.

	Crude OR (95% CI) ^a^	Adjusted OR (95% CI) ^b^	Adjusted OR (95% CI) ^b^	Adjusted OR (95% CI) ^c^
Single-exposure models				
Per 5-dBA increase in noise	1.64 (1.02–2.63) *	2.23 (1.26–3.93) *	—	2.97 (1.42–6.22) *
Per 1 μg/m^3^ increase in PM_10_	1.29 (0.49–3.38)	1.26 (0.45–3.54)	—	1.32 (0.46–3.78)
Per 1 ppb increase in NO_2_	0.80 (0.34–1.88)	0.82 (0.33–2.04)	—	0.78 (0.31–2.00)
Two-exposure model 1				
Per 5-dBA increase in noise	—		2.22 (1.26–3.93) *	2.96 (1.41–6.23) *
Per 1 μg/m^3^ increase in PM_10_	—		1.16 (0.40–3.38)	1.04 (0.36–3.04)
Two-exposure model 2				
Per 5-dBA increase in noise	—		2.22 (1.26–3.93) *	2.96 (1.41–6.23) *
Per 1 ppb increase in NO_2_	—		0.88 (0.34–2.26)	0.96 (0.37–2.49)

* *p* < 0.05. CVD, cardiovascular disease; dBA, A-weighted decibel; PM_10_, particulate matter less than 10 μm with aerodynamic diameter; NO_2_, nitrogen dioxides; OR, odds ratio; 95% CI, 95% confidence interval; ^a^ simple logistic regression models; ^b^ the multivariable logistic regression was adjusted for age, gender, body mass index, smoking, alcohol consumption, salt intake, regular exercise, and family history of hypertension; ^c^ the multivariable logistic regression was adjusted for age, gender, body mass index, smoking, alcohol consumption, salt intake, regular exercise, family history of hypertension, and total traffic flow rate.

## Abbreviations

CI, confidence interval; CVD, cardiovascular disease; dBA, A-weighted decibel; L_Aeq,8h_, 8-h-average A-weighted equivalent noise level; L_eq_, equivalent sound level; NO_2_, nitrogen dioxides; OR, odds ratio; PM_10_, particulate matter with dynamic diameter less than 10 μm; TWA, time-weighted average.
